# Incidencia de los problemas relacionados con los medicamentos antirretrovirales para el tratamiento de la infección por HIV en pacientes hospitalizados en el Hospital Santa Clara de Bogotá

**DOI:** 10.7705/biomedica.4345

**Published:** 2019-09-01

**Authors:** Carlos Andrés Badillo, Lizeth Katherine Barrera, Gerson Arias, Gabriel Fernando Tribiño, Óscar Andrés Gamboa, Julio César García, Ana María Granada

**Affiliations:** 1 Grupo de Investigación en Evidencia Terapéutica, Facultad de Medicina, Universidad de La Sabana, Chía, Colombia Grupo de Investigación en Evidencia Terapéutica Facultad de Medicina Universidad de La Sabana Chía Colombia; 2 Servicio de Infectología y Vigilancia Epidemiológica, Fundación Clinica Abood Shaio, Bogotá, D.C., Colombia Servicio de Infectología y Vigilancia Epidemiológica Fundación Clinica Abood Shaio BogotáD.C Colombia; 3 Posgrado de Farmacología Clínica, Facultad de Medicina, Universidad de La Sabana, Chía, Colombia Posgrado de Farmacología Clínica Facultad de Medicina Universidad de La Sabana Chía Colombia; 4 Grupo de VIH-TB, Hospital Santa Clara, Bogotá, D.C., Colombia Grupo de VIH-TB Hospital Santa Clara BogotáD.C Colombia

**Keywords:** VIH, terapia antirretroviral altamente activa, farmacovigilancia, efectos colaterales y reacciones adversas relacionadas con medicamentos, interacciones medicamentosas, errores de medicación, HIV, highly active antiretroviral therapy, pharmacovigilance, drug-related side effects and adverse reactions, drug interactions, medication errors

## Abstract

**Introducción.:**

El tratamiento antirretroviral de la infección por el virus de la inmunodeficiencia humana (HIV) se ha relacionado con diversos problemas de los medicamentos que causan o pueden causar la aparición de resultados negativos. En este contexto, es importante determinar su incidencia, caracterizarlos y clasificarlos para diseñar estrategias que minimicen su impacto.

**Objetivo.:**

Estimar la incidencia global y de cada uno de los problemas relacionados con los medicamentos antirretrovirales utilizados en el tratamiento del HIV en una cohorte de pacientes hospitalizados en una institución de tercer nivel de Bogotá.

**Materiales y métodos.:**

Se hizo un estudio descriptivo y retrospectivo de cohorte en pacientes de 18 años o más de edad con diagnóstico de infección por el HIV y en tratamiento antirretroviral, hospitalizados entre el 1° enero de 2015 y el 31 de diciembre de 2016 en el Hospital Santa Clara de Bogotá.

**Resultados.:**

La incidencia global de los problemas relacionados con los medicamentos antirretrovirales fue de 0,90 (IC95% 0,85-0,93). La incidencia de las interacciones medicamentosas fue de 0,85 (IC95% 0,80-0,90), la de las reacciones adversas de 0,28 (IC95% 0,22-0,35) y la del error de prescripción de 0,12 (IC95% 0,08-0,17).

**Conclusión.:**

Los problemas relacionados con los medicamentos deben estudiarse, diagnosticarse, prevenirse y tratarse para que el personal de salud pueda anticiparse a su aparición, disminuir su incidencia, implementar planes de manejo del riesgo y optimizar el cumplimiento del tratamiento antirretroviral.

## Abstract (en)

**Introduction:**

The antiretroviral therapy for the treatment of the human immunodeficiency virus (HIV) is a therapeutic strategy linked to drug-related problems that cause or can cause the appearance of negative results associated with the medication. It is important, therefore, to identify their incidence, characterize them, and classify them to design strategies to minimize their impact.

**Objective:**

To estimate the overall incidence and the incidence of each one of the antiretroviral drugs-related problems in the treatment of the HIV infection in a cohort of hospitalized patients in Bogotá, Colombia.

**Materials and methods:**

We conducted a descriptive, retrospective cohort study in patients aged 18 years or more diagnosed with HIV infection and under antiretroviral treatment hospitalized between January 1st, 2015, and December 31st, 2016, in the *Hospital Santa Clara*, Bogotá.

**Results:**

The overall incidence of antiretroviral drug-related problems was 0.90 (95% CI: 0.85-0.93). The incidence of drug-drug interactions was 0.85 (95% CI: 0.80-0.90), 0.28 (95% CI: 0.22-0.35) for adverse reactions, and 0.12 (CI 95%: 0.08-0.17) for prescription errors.

**Conclusion:**

Drug-related problems should be studied, diagnosed, prevented, and treated. Their knowledge can enable health care professionals to anticipate their emergence, reduce their incidence, implement risk management plans, and optimize adherence to antiretroviral treatment.

Desde su surgimiento en la década de los años 80, la epidemia originada por el virus de la inmunodeficiencia humana (*Human Immunodeficiency Virus*, HIV) ha sido una de la más graves de los tiempos modernos y uno de los mayores retos de la salud pública a nivel mundial, debido a la facilidad de su transmisión entre los seres humanos, la complejidad del tratamiento antirretroviral y la habilidad del virus para generar mecanismos de resistencia al tratamiento ^1^.

Según los datos epidemiológicos de la Organización Mundial de la Salud (OMS), en el 2017, 36,9 millones de personas estaban infectados por el HIV, de las cuales el 59 % se encontraba bajo tratamiento antirretroviral ^2^. En ese mismo año, hubo 1,8 millones de casos nuevos y 940.000 muertes asociadas con la infección por el HIV ^3^.

El tratamiento antirretroviral es una estrategia cuyos objetivos principales son reducir la morbilidad asociada con la infección por HIV, lograr y mantener la supresión de la viremia hasta valores indetectables, mejorar la calidad de vida de los pacientes, prolongar su supervivencia y disminuir el riesgo de transmisión del virus ^4^^,^^5^.

Hay cerca de 25 medicamentos para el tratamiento antirretroviral aprobados por la *Food and Drug Administration* (FDA) de los Estados Unidos ^4^^,^^5^. Sin embargo, la gran mayoría de estos medicamentos tienen una ventana terapéutica estrecha, con un amplio espectro de reacciones adversas, lo que, sumado a las frecuentes interacciones farmacológicas de los antirretrovirales con otros fármacos y el número de errores de medicación, implica múltiples problemas relacionados con estos medicamentos en la práctica clínica diaria. Las reacciones adversas indeseables relacionados con el tratamiento antirretroviral pueden conducir a la aparición de resultados negativos, como el cumplimiento deficiente del tratamiento, la resistencia a los antirretrovirales y el incremento de los costos por recaídas y fallas terapéuticas ^6^.

Los resultados negativos pueden ser producto de los problemas relacionados con los medicamentos, los reacciones adversas, los errores de medicación y las interacciones medicamentosas ^6^. Los problemas relacionados con los medicamentos competen a la farmacovigilancia, cuyo objetivo es mejorar la atención del paciente fomentando el uso seguro y racional de los medicamentos ^7^, por lo que esta constituye una herramienta indispensable para la prevención de los riesgos asociados con el uso de medicamentos y la disminución de su impacto negativo en la salud humana mediante planes de manejo de los riesgos y reducción de los altos costos en salud que dichos problemas generan.

El tratamiento antirretroviral ha permitido que la expectativa de vida de los pacientes con infección por HIV aumente, con el resultado de que las comorbilidades asociadas con el envejecimiento, como la diabetes mellitus, la hipertensión arterial sistémica o las dislipidemias se incrementen, así como aquellas propias de la infección por HIV, como las neoplasias y las infecciones oportunistas ^8^. Los pacientes que reciben el tratamiento son de especial interés desde el punto de vista farmacológico dado el uso de múltiples medicamentos y sus efectos potenciales en la seguridad y la efectividad clínicas, por lo que se les considera pacientes en alto riesgo de desarrollar problemas relacionados con los medicamentos ^9^.

La gestión del riesgo asociado con dichos problemas en el tratamiento antirretroviral requiere determinar la incidencia total y las de cada uno de los problemas por grupo poblacional, si es factible. En este estudio, se hizo una revisión retrospectiva de una cohorte de pacientes con infección por HIV bajo tratamiento antirretroviral, hospitalizados en el Hospital Santa Clara de Bogotá durante el 2015 y el 2016. Este es un hospital de tercer nivel que coordina un programa de referencia en el país para pacientes con infección por HIV. Los resultados del estudio brindan herramientas para ofrecer mejor seguridad y efectividad en el tratamiento de la infección por HIV, disminuyendo las fallas terapéuticas, los reingresos hospitalarios y la necesidad de los tratamientos para combatir las complicaciones.

## Materiales y métodos

Se hizo un estudio descriptivo y retrospectivo de cohorte con pacientes de 18 años de edad o más con diagnóstico confirmado de infección por HIV y bajo tratamiento antirretroviral, hospitalizados por cualquier causa entre el 1° enero de 2015 y el 31 de diciembre de 2016 en el Hospital Santa Clara de Bogotá.

Este es un hospital público de tercer nivel de atención adscrito a la Secretaría de Salud de Bogotá, que tiene convenios de docencia con universidades para la práctica médica. La zona geográfica de influencia del hospital incluye todo el Distrito Capital por ser centro de referencia en neumología, cirugía cardiovascular y hemodinámica, salud mental, toxicología, y cuidado crítico pediátrico y en adultos. Cuenta con profesionales de la salud especializados en la atención de pacientes con infección por HIV en el servicio de urgencias, en el de consulta externa y durante la hospitalización ^10^.

Los datos se recolectaron y se organizaron en una base de datos creada por los investigadores en Epidata, versión 2.0.10.26, que incluyó las variables analizadas.

El estudio estimó la incidencia total y de cada uno de los problemas relacionados con los medicamentos antirretrovirales (reacciones adversas, errores de medicación, interacciones medicamentosas) utilizados en el tratamiento de la infección por HIV.

Además, los reacciones adversas detectados se clasificaron según el sistema orgánico comprometido y la seriedad que revestían; también, se analizó la causalidad y se detallaron los fármacos implicados; se establecieron los errores de prescripción según los criterios de la *American Society of Health- System Pharmacists* (ASHP). Se caracterizaron las interacciones detectadas en términos de los fármacos implicados, el mecanismo de producción y la seriedad potencial de la interacción con el programa Lexicomp™ (Wolters Kluwer Clinical Drug Information, OH, USA). Por último, se estimó de manera exploratoria la incidencia de los problemas relacionados con los medicamentos por grupos de edad, por sexo y por comorbilidades.

Para el cálculo del tamaño de muestra, se incluyeron los pacientes que cumplían con los criterios de selección a partir del 1° de enero de 2015. A medida que se fueron incluyendo los pacientes, se estimaron las incidencias con sus intervalos de confianza. Se suspendió el reclutamiento cuando se alcanzó una precisión de ± 5 % en la incidencia estimada, lo que se logró con 200 pacientes. El periodo de estudio se cerró el 31 de diciembre de 2016 con 204 pacientes.

### Reacciones adversas de los medicamentos

Para establecer las reacciones adversas, se utilizaron las *Guidelines for the use of antiretroviral agents in HIV-1-infected adults and adolescents*, *Department of Health and Human Services*^11^ de los *National Institutes of Health* (NIH) de los Estados Unidos, y se agruparon según el sistema orgánico comprometido (reacciones adversas gastrointestinales, cutáneas y neurológicas, entre otras), de tal manera que su detección fuera más fácil para el personal de salud y, luego, se clasificaron según el grupo farmacológico antirretroviral implicado (inhibidores de la transcriptasa inversa nucléosidos, no nucléosidos y nucleótidos, inhibidores de la proteasa, inhibidores de la integrasa, inhibidores de fusión y antagonistas del correceptor CCR5).

Además, se evaluó la relación de causalidad de las reacciones adversas utilizando el algoritmo de Naranjo, *et al.,* con el cual se determina si una reacción es causada por el uso de un medicamento y no es el resultado de otros factores, mediante una serie de preguntas basadas en los criterios de Bradford Hill para la búsqueda de relaciones causales ^12^.

La ventaja más importante de este algoritmo frente a otros algoritmos o métodos de evaluación de la causalidad es su menor propensión a las variaciones subjetivas, comparado con el sistema de la OMS y el *Uppsala Monitoring Centre* (UMC) basado en el criterio de expertos. Asimismo, es ampliamente aceptado y utilizado en todo el mundo y consume menos tiempo que otros algoritmos ^12^^-^^14^.

### Errores de prescripción

Los errores de prescripción se evaluaron con base en la historia clínica, ya que en ella se encontraba registrada la información sobre todos los medicamentos incluidos en el tratamiento. Las prescripciones halladas se compararon con las recomendaciones para el uso de los fármacos antirretrovirales publicadas en la “Guía de práctica clínica basada en la evidencia científica para la atención de la infección por HIV/sida en adolescentes (con 13 años de edad o más) y adultos” del Ministerio de Salud y Protección Social de Colombia, publicada en el 2014 ^15^.

### Interacciones medicamentosas

Para el análisis de las interacciones fármaco-fármaco (antirretrovirales y fármacos de uso concomitante), se emplearon los datos prescriptivos extraídos de las historias clínicas y el programa Lexicomp™, sistema de análisis capaz de evaluar posibles interacciones entre los medicamentos y clasificarlas según la gravedad con bases de datos comprobados (cuadro 1).

**Cuadro 1:** Clasificación de riesgo de la interacción según el programa Lexicomp™

Clasificación de riesgo de la interacción	Comentario
X: evitar la combinación	La evidencia demuestra que los agentes específicos pueden interactuar con un impacto clínico demostrado. El riesgo asociado del uso concomitante de estos agentes usualmente sobrepasa los beneficios. Se considera contraindicación.
D: contemplar la modificación del tratamiento	La evidencia demuestra que los agentes específicos pueden interactuar con un impacto clínico demostrado. Se debe evaluar individualmente para determinar si los beneficios superan los riesgos. Se debe supervisar el tratamiento, cambiar la dosis o usar agentes alternativos.
C: supervisar el tratamiento	La evidencia demuestra que los agentes específicos pueden interactuar con un impacto clínico demostrado. Los beneficios del uso concomitante de los fármacos usualmente superan los riesgos. Un plan de supervisión apropiado debe implementarse para determinar potenciales efectos secundarios.
B: no se requiere acción	Los datos demuestran que los agentes específicos pueden interactuar, sin embargo, son pocos en cuanto a su impacto clínico.
A: interacción no conocida	La evidencia no ha demostrado interacciones farmacocinéticas o farmacodinámicas entre los agentes implicados.

Dicho programa provee información clara y precisa relacionada con los medicamentos (dosis, administración, precauciones y advertencias), y con el componente clínico (guías de manejo e interacciones medicamentosas). Además, esta base de datos se actualiza diariamente, ofreciendo así información científica adecuada y aumentando la sensibilidad en la búsqueda de potenciales interacciones medicamentosas. Se ha empleado en distintos estudios en los que se evaluaron las potenciales interacciones medicamentosas.

El uso de Lexicomp™ exige comprar una licencia que, para este estudio, se obtuvo mediante la base de datos electrónica de la biblioteca Octavio Arizmendi Posada de la Universidad de La Sabana.

### Análisis de datos

Se hicieron análisis descriptivos usando medidas de tendencia central (media, mediana), ubicación (percentiles) y dispersión (desviación estándar y rangos) para las variables cuantitativas.

En la descripción de las variables cualitativas se usaron frecuencias absolutas y relativas. Se calculó la incidencia, con un intervalo de confianza del 95 %, de los problemas relacionados con los medicamentos: número de eventos (primer evento) observado frente al total de la población en riesgo durante el periodo de observación, así como la densidad de la incidencia de las reacciones adversas, con un intervalo de confianza del 95 %, calculándola con respecto al total de personas-día; también, se estableció la incidencia según el tipo de problema relacionado con los medicamentos.

Asimismo, dado que un paciente puede presentar más de uno de dichos problemas, se estimó el promedio de cada uno de ellos por persona, con su intervalo de confianza del 95 %, así como el total de eventos observados con respecto a la población en riesgo durante el periodo de observación.

Por otra parte, se acogieron los siguientes criterios de seriedad de las reacciones adversas ^7^^,^^16^: serias, los que implicaban muerte, amenaza para la vida, prolongación de la hospitalización, incapacidad o discapacidad permanente o significativa, y no serias, aquellos que no cumplían con los criterios de seriedad.

Por último, se estableció la relación de causalidad de las reacciones adversas ^12^.

### Consideraciones éticas

El estudio se ajustó a los contenidos éticos de la Declaración de Helsinki de la Asociación Médica Mundial, así como a los de la Resolución 8430 de 1993 del Ministerio de Salud de Colombia, en la cual este tipo de estudio se clasifica como “investigación sin riesgo”.

Se hizo una revisión documental de las historias clínicas protegiendo la confidencialidad, pues no se identificó a los sujetos (en la base de datos no se registraron nombres ni números de documento), y se tomaron medidas para suprimir los datos de identidad de los sujetos. No se hicieron intervenciones clínicas durante el estudio y, por lo tanto, no hubo exposición de los pacientes a factores de riesgo.

El estudio estuvo a cargo de personal clínica y éticamente idóneo. Los investigadores principales asumieron la responsabilidad completa por el trabajo y declararon no tener ninguna clase de conflictos de intereses. Para el manejo de los datos se tuvo en cuenta el artículo 6 de la Ley Estatutaria 1581 de 2012.

## Resultados

### Características poblacionales

**Figura 1:**
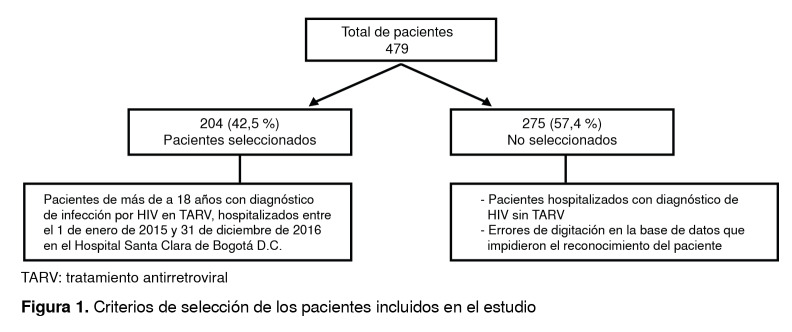
Criterios de selección de los pacientes incluidos en el estudio

En la base de datos se incluyeron 479 pacientes hospitalizados con diagnóstico confirmado de infección por HIV en el período comprendido entre el 1° de enero de 2015 y el 31 de diciembre de 2016, de los cuales 204 cumplían con los criterios de selección (figura 1). La mayoría de ellos eran de sexo masculino (n=157, 77 %), con una edad promedio de 40 años ± 11 (rango de 18 a 84 años), y la relación hombre a mujer fue de 3,3 a 1 (cuadro 2).

**Cuadro 2:** Características demográficas y clínicas de los pacientes

Características demográficas	Población (n=204)
Edad (años)	
Media	40 ± 11
Rango	18-84
Sexo	n (%)
Masculino	157 (77)
Femenino	47 (23)
Estrato socioeconómico*	
1	14 (7)
2	4 (2)
Sin información	186 (91)
Nivel educativo	
Ninguno	2 (1)
Primaria	10 (5)
Secundaria	21 (10)
Técnico	2 (1)
Universitario	1 (0,5)
Sin información	168 (82,5)

* El estrato socioeconómico en Colombia se clasifica del 1 a 6, en donde 1 es el más bajo y 6 el más alto

**Table:** 

Características demográficas	Población (n=204)
Recuento de linfocitos T CD4	
Media	121 células/ml
Rango	0-793 células/ml
Clasificación de la infección por HIV según clasificación del CDC, 2014	n (%)
A1	2 (1)
A2	2 (1)
A3	8 (4)
B1	0 (0)
B2	3 (1,5)
B3	4 (2)
C1	1 (0,5)
C2	4 (2)
C3	144 (70,5)
Sin información	36 (17,5)
Consumo de sustancias psicoactivas	
Sí	65 (32)
No	121 (59)
Sin información	18 (9)
Esquema de tratamiento antirretroviral	
ITR nucleósido-nucleótido más inhibidor de la integrasa	20 (10)
ITR nucleósido-nucleótido más ITR no nucleósido	97 (47)
IP más ITR nucleósido-nucléotido	78 (38)
Otros esquemas	9 (5)
Comorbilidades	
Enfermedad infecciosa	121 (59)
Enfermedad psiquiátrica	21 (10)
Enfermedad neurológica	18 (9)
Enfermedad hematológica	12 (6)
Otras	32 (16)
Función renal (TFG según Cockroft Gault)	
<15 ml/minuto/1,73 m2	3 (1,5)
16-29 ml/minuto/1,73 m2	3 (1,5)
30-59 ml/minuto/1,73 m2	21 (10,3)
60-89 ml/minuto/1,73 m2	39 (19,1)
>90 ml/minuto/1,73 m2	60 (29,4)
Sin información	78 (38,2)
Presencia de disfunción hepática	
Sí	9 (4,4)
No	195 (95,6)

ITR: inhibidor de la transcriptasa inversa ; IP: inhibidor de proteasa; TFG: tasa de filtración glomerular; CDC: Centers for Disease Control and Prevention

Según la clasificación de los *Centers for Disease Control and Prevention* (CDC) de los Estados Unidos para el 2014, el 70,5 % (n=144) de los pacientes se encontraba en el estadio C3 de la enfermedad, con un promedio de recuento de linfocitos T CD4 (LTCD4) de 121 células/ml.

Se determinaron los diferentes tratamientos antirretrovirales usados en los pacientes estudiados (cuadro 3), y la distribución por grupos de antirretrovirales indicó que el esquema basado en inhibidores de la transcriptasa inversa nucleósidos-nucleótidos más no nucleósidos fue el más empleado (47 % de los pacientes), seguido del esquema basado en inhibidores de la proteasa más inhibidores de la transcriptasa inversa nucleósidos-nucleótidos (38 % de los pacientes). Los antirretrovirales más empleados en cada esquema de tratamiento fueron lamivudina, zidovudina, fumarato de disoproxilo de tenofovir-emtricitabina, efavirenz, lopinavir- ritonavir y atazanavir-ritonavir. No se registraron esquemas de tratamiento con inhibidores del correceptor CCXR-5 ni con fármacos inhibidores de la fusión.

**Cuadro 3:** Tratamientos antirretrovirales usados en los pacientes estudiados

Grupo terapéutico	Fármaco antirretroviral
ITR nucleósido	Abacavir
	Zidovudina
	Lamivudina
	Didanosina
ITR no nucleósido	Efavirenz
	Nevirapina
	Rilpivirina
ITR nucleótido	Tenofovir
Inhibidores de la proteasa	Lopinavir-ritonavir
	Atazanavir-ritonavir
	Darunavir-ritonavir
	Fosamprenavir-ritonavir
Inhibidores de la integrasa	Raltegravir
	Dolutegravir

ITR: inhibidor de la transcriptasa inversa

También, se estableció que 59,3 % de los pacientes presentaba alguna comorbilidad de tipo infeccioso, 10,2 % tenía una comorbilidad psiquiátrica y 8,8%, una enfermedad neurológica. Además, se evidenció que 32 % de la población evaluada presentaba antecedentes de consumo de sustancias psicoactivas.

### Incidencia de los problemas relacionados con los medicamentos

La incidencia total de estos problemas (reacciones adversas, errores de medicación e interacciones medicamentosas) fue de 0,90 (IC95% 0,85-0,93). Al evaluar los datos según el tipo de problema específico, se evidenció una incidencia de 0,85 (IC95% 0,80-0,90) de la interacción medicamentosa, de 0,28 (IC95% 0,22-0,35) de las reacciones adversas, y una de 0,12 (IC95% 0,08-0,17) de los errores de prescripción. Asimismo, la densidad de la incidencia de las reacciones adversas fue de 9 por 100 personas-día de hospitalización.

De manera exploratoria, se estableció que la incidencia de los problemas relacionados con los medicamentos según el sexo fue de 0,91 (IC95% 0,86- 0,95) en hombres y de 0,85 (IC95% 0,71-0,93) en mujeres, en tanto que por grupo etario fue de 0,95 (IC95% 0,90-0,98) para los adultos entre los 20 y los 39 años, de 0,84 (IC95% 0,74-0,91) para la población entre los 40 y los 64 años, y de 0,83 (IC95% 0,35-0,99) para la población mayor de 65 años.

La incidencia por subgrupo de comorbilidades fue de 0,97 (IC95% 0,92-0,99) para infecciones, 0,91 (IC95% 0,61-0,99) para enfermedades cardiorrespiratorias y de 0,76 (IC95% 0,60-0,88) para condiciones neuropsiquiátricas.

### Reacciones adversas de los medicamentos

Se documentaron 73 reacciones adversas, con un promedio de 0,35 episodios por persona (IC95% 0,26-0,43); el sistema gastrointestinal fue el más afectado (58 %; n=42), seguido del hematológico (11 %; n=8), el neurológico (n=5) y el cardiovascular (n=5), ambos con el 7 %. Al discriminar las principales reacciones adversas que comprometían el sistema gastrointestinal, se estableció que el 45 % del total correspondió a náuseas, 28,5 % a diarrea y 19 % a vómito. Por otro lado, las principales reacciones adversas en el sistema hematológico fueron anemia, en el 75 %, y neutropenia, en el 25 % de los casos. La cefalea (60 %), el vértigo (20 %) y el insomnio (20 %) fueron las más frecuentes en el sistema neurológico. La taquicardia fue la reacción adversa más detectada en el sistema cardiovascular (80 % de los casos).

El grupo de medicamentes más frecuentemente implicado en las reacciones adversas fue el de los inhibidores de la transcriptasa inversa nucleósidos, en el 56 % de los casos, seguido de los inhibidores de la proteasa, en el 23 %, seguidos por los inhibidores de la transcriptasa inversa nucleótidos, en el 12 %, los inhibidores de la transcriptasa inversa no nucleósidos, en el 7 %, y los inhibidores de la integrasa en el 2 %. Asimismo, se determinaron los fármacos antirretrovirales más frecuentemente implicados en las reacciones adversas (cuadro 4).

**Cuadro 4:** Fármacos implicados en los efectos secundarios a medicamentos

Antirretroviral	Efectos secundarios asociados (n=73) n (%)
Zidovudina-lamivudina	23 (31,5)
Tenofovir-emtricitabina	18 (24,6)
Lopinavir-ritonavir	13 (17,8)
Abacavir-lamivudina	11 (15)
Atazanavir- itonavir	4 (5,4)
Efavirenz	4 (5,4)

Al evaluar la causalidad de las reacciones adversas con el algoritmo de Naranjo, *et al.,* se encontró que del total (n=73), el 93,1 % tuvo una asociación causal posible, 4,11 %, una probable, y 2,74 %, una definitiva. Además, 52 (71,2 %) de los 73 determinados se clasificaron como no serios y 21 (28,8 %) como serias.

### Errores de prescripción

Se documentaron 30 errores de dosificación, con un promedio de 0,14 errores por persona (IC95% 0,08-0,20). No se documentaron errores por selección incorrecta del medicamento prescrito, forma farmacéutica o vía de administración. De los errores evaluados, los relacionados con los inhibidores de la transcriptasa inversa nucleósidos-nucleótidos fueron los más frecuentes (n=24), especialmente la lamivudina y el fumarato de disoproxilo de tenofovir, seguido por los relacionados con los inhibidores de la proteasa (n=5) y con inhibidores de la transcriptasa inversa no nucleósidos (n=1). La mayoría de los errores de prescripción se debieron a no haber ajustado la dosis según la función renal (cuadro 5), siendo la lamivudina el principal medicamento implicado (53,3 % de los casos), seguida del fumarato de disoproxilo de tenofovir-emtricitabina (36,6 % de los casos).

### Interacciones medicamentosas

Se evaluaron 714 interacciones medicamentosas, con un promedio de 3,4 (IC95% 3,12-3,86) interacciones por paciente. El 85,7 % de los pacientes presentó, al menos, una interacción y el 73,5 % presentó dos o más interacciones medicamentosas. Cabe mencionar que 9 pacientes (4,4 %) presentaron 10 interacciones.

**Cuadro 5:** Ejemplos de errores de dosificación

Medicamento	Ajuste	Error
Lamivudina	150 mg al día	300 mg/día en falla renal con TFG de 34 ml/minuto/1,73 m2
Lamivudina	150 mg al día	300 mg cada 8 horas con TFG de 47 ml/minuto/1,73 m2
Lamivudina	150 mg/día y luego 100 mg/día	300 mg/día con TFG de 21 ml/minuto/1,73 m2
Tenofovir	No formular	Se formuló en lesión renal aguda
Tenofovir	300 mg cada 48 h	300 mg cada día con TFG de 41 ml/minuto/1,73 m2
Tenofovir	300 mg cada 72 h	300 mg c/día con TFG de 21 ml/minuto/1,73 m2
Emtricitabina	200 mg cada 48 h	200 mg/día
Nevirapina	200 mg cada 12 h	200 mg/día
Didanosina	125 mg/día	400 mg/día

TFG: tasa de filtración glomerular

### Interacciones medicamentosas

Se evaluaron 714 interacciones medicamentosas, con un promedio de 3,4 (IC95% 3,12-3,86) interacciones por paciente. El 85,7 % de los pacientes presentó, al menos, una interacción y el 73,5 % presentó dos o más interacciones medicamentosas. Cabe mencionar que 9 pacientes (4,4 %) presentaron 10 interacciones.

Los grupos farmacológicos implicados en las interacciones farmacológicas fueron los inhibidores de la transcriptasa inversa no nucleósidos (n=296, 41,4 %), los inhibidores de la proteasa (n=265, 37,1 %), los inhibidores de la transcriptasa inversa nucleósidos-nucleótidos (n=134, 18,7 %) y los inhibidores de la integrasa (n=19, 2,6 %).

**Figura 2:**
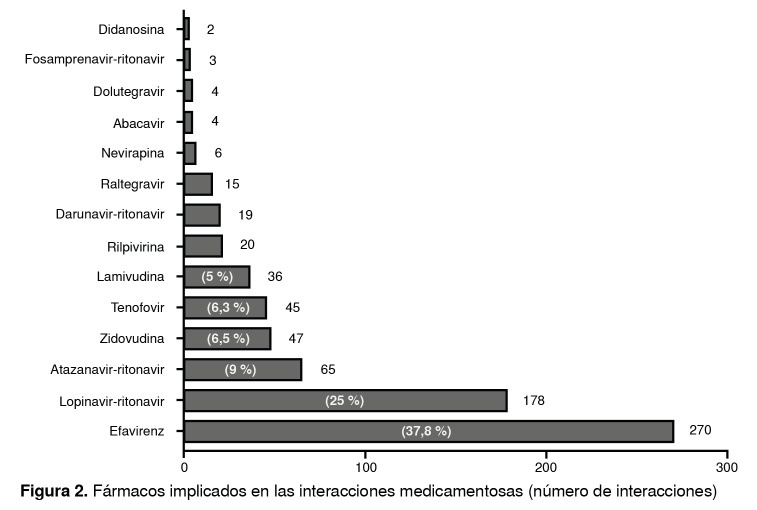
Fármacos implicados en las interacciones medicamentosas (número de interacciones)

El efavirenz fue el inhibidor de la transcriptasa inversa no nucleósido con mayor número de interacciones (37,8 % del total), seguido por la combinación de lopinavir-ritonavir y de atazanavir-ritonavir con 25 y 9 %, respectivamente (figura 2). A pesar de que la zidovudina, el fumarato de disoproxilo de tenofovir y la lamivudina se utilizaron frecuentemente en el tratamiento antirretroviral en la población de estudio, su porcentaje de interacciones fue bajo: 6,5, 6,3 y 5 %, respectivamente.

Los principales fármacos con los cuales interactuaron los inhibidores de la transcriptasa inversa no nucléosidos, fueron el trimetoprim-sulfametoxazol (22,2 %), el fluconazol (9,6 %) y la trazodona (9,2 %). Por otro lado, las interacciones más frecuentes observadas con los inhibidores de la proteasa se presentaron con trimetoprim-sulfametoxazol (16,6 %), omeprazol (12 %), fluconazol (10 %) y claritromicina (6,4 %). Las principales interacciones de la zidovudina se dieron con claritromicina (27,6 %), fluconazol (25,5 %) y dipirona (21,2 %). El 100 % de las interacciones de la lamivudina fueron con el trimetoprim-sulfametoxazol, en tanto que el 40 % de las interacciones del fumarato de disoproxilo de tenofovir fueron con aciclovir y, en menor porcentaje, con dipirona (31,1 %).

Según la clasificación mecánica de las interacciones, el 50,5 % fue de tipo farmacodinámico y el 49,5 %, de tipo farmacocinético.

Según la herramienta Lexicomp™, el 47,7 % (n=341) de las 714 interacciones se categorizó como de categoría C (necesidad de supervisar el tratamiento), seguidas de las de categoría D (contemplar la modificación del tratamiento) con 35 % (n=250), de categoría X (evitar la combinación) con 10,6 % (n=76) y de categoría B (sin necesidad de intervención) con un 6,5 % (n=47).

Al analizar las interacciones categorizadas como X, se encontró que se debían principalmente al uso de lopinavir-ritonavir con claritromicina (riesgo de prolongación del intervalo QT) en 17 % de los casos, seguido de la combinación de zidovudina con dipirona (potenciación del efecto tóxico mielosupresor de ambos medicamentos con incremento en el riesgo de desarrollar agranulocitosis y pancitopenia) y de la combinación de lopinavir- ritonavir con trazodona (riesgo de prolongación del intervalo QT), ambas combinaciones en un 13 % de los casos. La interacción entre lopinavir-ritonavir y metronidazol correspondió al 12 % seguida de la de lopinavir-ritonavir con ciprofloxacina con el 10,5 %, ambas con riesgo de prolongar el intervalo QT.

Al analizar las interacciones de tipo D, se evidenció que las más frecuentes se debieron a las combinaciones de lopinavir-ritonavir con trimetoprim-sulfametoxazol (n=30, 12 %), lopinavir-ritonavir con fluconazol (n=19, 7,6 %) y atazanavir-ritonavir con omeprazol (n=15, 6 %).

## Discusión

Actualmente existen pocos estudios en el país y en Latinoamérica en que se haya evaluado la incidencia de los problemas relacionados con los medicamentos, lo que indicaría la falta de interés en torno a su impacto en la atención hospitalaria. Los resultados del presente estudio evidenciaron que el 90 % de los pacientes presentaron dichos problemas, lo cual indica el alto riesgo de la población hospitalizada que está bajo tratamiento antirretroviral de presentar resultados negativos asociados con este y la importancia de generar mecanismos que gestionen el riesgo en aras de su seguridad.

Si bien la incidencia de los problemas relacionados con los medicamentos fue similar en los grupos de edad evaluados, es importante tener precaución y hacer hincapié en los pacientes adultos mayores, ya que usualmente toman múltiples fármacos, lo que suele incrementar el riesgo de presentar tales problemas, por ejemplo, reacciones adversas e interacciones medicamentosas, así como el aumento en las hospitalizaciones y el poco cumplimiento del tratamiento ^17^.

Llamó la atención la mayor incidencia de los problemas relacionados con los medicamentos en el análisis del subgrupo de pacientes con infecciones concomitantes, lo que responde, en gran parte, a que estas fueron la comorbilidad más frecuente, aunque también se asociaría con una mayor probabilidad de interacciones entre los antirretrovirales y los fármacos contra las infecciones, por ejemplo, claritromicina, rifampicina y fluconazol, entre otros. En este sentido, es necesario proveer antimicrobianos de menor interacción con el tratamiento antirretroviral, por ejemplo, azitromicina o rifamicina, entre otros.

Según los resultados, el 28 % de los pacientes evaluados había presentado alguna reacción adversa, porcentaje que se encuentra en el rango de los valores reportados en la literatura, los cuales oscilan entre el 4 y el 85 % ^18^^-^^20^; esta variabilidad se atribuye a la heterogeneidad de las poblaciones y los tratamientos evaluados en los estudios, como la raza, el periodo de seguimiento, la hospitalización y los esquemas antirretrovirales empleados. Esto resulta de especial interés al considerar que las reacciones adversas son una reacción nociva y no intencionada a un fármaco ^21^^,^^22^, y que algunos son prevenibles, por lo que es pertinente implementar un sistema activo de farmacovigilancia, así como programas de educación médica para garantizar la seguridad de los pacientes.

Más de la mitad de las reacciones adversas afectaron el sistema gastrointestinal, y las náuseas, la diarrea y el vómito fueron las más frecuentes. Otros sistemas afectados, aunque en menor proporción, fueron el sistema hematológico y el neurológico. La densidad de la incidencia de las reacciones adversas en el estudio respalda la necesidad de hacer un seguimiento activo, constante y periódico a la población hospitalizada que recibe tratamiento antirretroviral para detectar tempranamente posibles reacciones adversas.

En otros estudios se han reportado datos similares a los encontrados en este. Es el caso del estudio de cohorte prospectivo de Bezabhe, *et al.,* en Etiopía, en el cual se evaluaron 211 pacientes y se registró una densidad de la incidencia de reacciones adversas de 14,8 por 100 personas-mes y el 54,5 % de pacientes con reacciones adversas gastrointestinales, y las náuseas y el vómito fueron las más frecuentes, seguidas por el 53,5 % de reacciones adversas neuropsiquiátricos ^19^.

Por otra parte, en un estudio analítico observacional publicado en el 2007 que se llevó a cabo en una cohorte histórica en un hospital de Lima, se reportó una densidad de incidencia de 9,1 por 10 personas-año de seguimiento; el sistema gastrointestinal estuvo comprometido en 28,5 % del total de las reacciones adversas detectadas, seguido por el sistema hematológico en el 23,4 % ^18^^,^^19^. El esquema de zidovudina-lamivudina fue el más frecuentemente asociado con las reacciones adversas, seguido por el de fumarato de disoproxilo de tenofovir-emtricitabina y lopinavir-ritonavir. El impacto de estos hallazgos en la práctica clínica exigiría una mayor vigilancia y mejor seguimiento de los pacientes, con el fin de mejorar el cumplimiento del tratamiento y su seguridad.

En los estudios dedicados a determinar errores de medicación se ha determinado que ocurren con mayor frecuencia en el momento de prescribir el tratamiento y se relacionan con la dosis, la frecuencia, las posibles interacciones con medicamentos y con alimentos, los esquemas incompletos y con el hecho de no ajustar la dosis en condiciones especiales como, por ejemplo, la falla renal.

Al analizar los errores de dosificación, se evidenció que el 12 % de los pacientes presentaba este tipo de problema, la mayoría por no ajustar la dosis teniendo en cuenta la disfunción renal, principalmente de lamivudina y fumarato de disoproxilo de tenofovir. Es posible que dicha incidencia se haya subestimado, ya que la historia clínica de la gran mayoría de los pacientes no registraba datos sobre la función renal. Ello repercute en un mayor riesgo de toxicidad para los pacientes debido al incremento del tiempo de vida media plasmática de los medicamentos condicionado por la disminución de la tasa de filtración glomerular.

Por otra parte, si bien se encontraron pacientes con disfunción hepática (lo cual incide en el ajuste de la dosis y, consecuentemente, puede constituir un error de prescripción), no fue posible caracterizar el grado de disfunción hepática para determinar si se requería el ajuste de la dosis porque no todos los datos se encontraban en las historias clínicas revisadas.

Los errores de medicación son prevalentes en los pacientes hospitalizados con diagnóstico de infección por HIV ^23^. En un estudio retrospectivo de Rastegar*, et al.*, en el cual se evaluaron los errores de medicación en una cohorte de 209 pacientes con diagnóstico de infección por HIV, el error más frecuente fue el relacionado con la cantidad o la frecuencia de las dosis (16,3 % de los pacientes), seguido por el de administrar un antirretroviral con otro medicamento contraindicado ^24^.

Por otro lado, en un estudio publicado en *Annals of Pharmacotherapy*^25^ en el 2008 se documentó que había habido al menos un error de medicación en el 72 % de los pacientes evaluados y que el 56 % de estos errores tenía el potencial de causar deterioro clínico.

Si bien en el presente estudio no se documentaron tantos errores como en el de 2008, los datos ameritan un llamado de alerta frente a la necesidad de evaluar de manera periódica y minuciosa las formulaciones de los pacientes. Se ha documentado suficientemente que los problemas relacionados con los medicamentos conllevan consecuencias clínicas importantes en este grupo específico de pacientes, como la aparición de resistencia viral, la toxicidad farmacológica y el deficiente control sintomático, lo que se traduce en el aumento de los costos para el sistema de salud, la prolongación de la hospitalización e, inclusive, el aumento en la mortalidad ^7^^,^^17^^,^^26^.

En cuanto a las interacciones farmacológicas, los resultados del presente estudio evidenciaron una incidencia de problemas relacionados con los medicamentos del 85 %, lo cual refleja la complejidad del tratamiento antirretroviral. Incluso, se documentaron casos de pacientes que presentaron hasta diez interacciones, lo que representa un incremento en el riesgo de seguridad y la eficacia de los medicamentos implicados. En este sentido, resulta explicable que los inhibidores de la transcriptasa inversa no nucleósidos y los de la proteasa sean los de mayor potencial de interacción, pues tienen la facultad de inducir e inhibir distintas enzimas del sistema microsómico P450, por ejemplo, los citocromos CYP3A4, CYP2C9, y CYP2C19, entre otros ^11^.

En la caracterización de las interacciones medicamentosas se registró un porcentaje no despreciable de interacciones de tipo X (10,6 %), las cuales pueden tener resultados clínicos adversos graves y, por lo tanto, pueden requerir un cambio en el tratamiento, por ejemplo, para evitar la interacción entre zidovudina y dipirona podría optarse por otro tipo de analgésico para prevenir el riesgo de agranulocitosis. Además, en las interacciones entre lopinavir-ritonavir y antibióticos y antidepresivos es de vital importancia monitorizar periódicamente el intervalo QT en caso de no poder cambiar el tratamiento.

En el presente estudio se encontraron interacciones medicamentosas con gran potencial de riesgo de resultados negativos, lo cual coincide con lo reportado en el estudio de Machado*, et al.,* en Colombia, quienes documentaron 49 interacciones entre los fármacos antirretrovirales y los medicamentos administrados concomitantemente con riesgos potenciales, siendo los inhibidores de la proteasa los implicados en 40 % de estas asociaciones. La interacción más frecuente se dio entre estos inhibidores y las estatinas (49 %), y la de estos y los antidepresivos (10,2 %) ^27^.

Los hallazgos de este estudio resaltan la importancia de contar con el conocimiento necesario para detectar los principales problemas relacionados con medicamentos, disminuir su incidencia, optimizar el cumplimiento del tratamiento antirretroviral entre los pacientes y, probablemente, su efectividad al disminuir las interacciones medicamentosas y la aparición de reacciones adversas y, además, disminuir los costos directos derivados de la atención en salud para enfrentar tales problemas, así como las complicaciones generadas por el incumplimiento del tratamiento antirretroviral.

Las limitaciones del estudio incluyen su naturaleza retrospectiva, pues no se contó con los elementos necesarios para establecer con certeza la relación de causalidad de las reacciones adversas; además, solo se evaluó un error de medicación (en la prescripción) y no se evaluó el impacto clínico de las interacciones medicamentosas. Sin embargo, el estudio constituye un punto de partida para futuros análisis orientados a la farmacovigilancia del tratamiento antirretroviral de la infección por el HIV, así como para el diseño de estrategias institucionales encaminadas a mejorar la seguridad y disminuir los riesgos derivados del uso de los medicamentos antirretrovirales mediante recomendaciones prescriptivas y de seguimiento, y de programas de educación para los profesionales de la salud.

El estudio aportó datos sobre la incidencia de los problemas relacionados con los medicamentos en pacientes hospitalizados con diagnóstico de infección por HIV y bajo tratamiento antirretroviral. Tales problemas son frecuentes en este tipo de pacientes y, por lo tanto, deben estudiarse, diagnosticarse, prevenirse y tratarse en el marco de planes de manejo del riesgo que maximicen los beneficios y minimicen los riesgos de los fármacos. Los inhibidores de la transcriptasa inversa nucleósidos fueron los fármacos más implicados en las reacciones adversas, y el sistema gastrointestinal fue el más afectado. La mayoría de los errores de prescripción se debieron a que no se ajustó la dosis según la función renal de los pacientes. La gran cantidad de interacciones medicamentosas demuestra la complejidad del tratamiento antirretroviral y su potencial de causar resultados negativos en los pacientes. El conocimiento sobre los principales problemas relacionados con los medicamentos en pacientes bajo tratamiento antirretroviral permite disminuir su incidencia, optimizar el cumplimiento del tratamiento y mejorar su efectividad.

Asimismo, el estudio contribuye y refuerza la farmacovigilancia del tratamiento antirretroviral, por lo que se recomienda contar con un especialista en farmacología clínica en la atención de este grupo de pacientes.
